# Investigating the Effects of Microclimate on Arboviral Kinetics in *Aedes aegypti*

**DOI:** 10.3390/pathogens13121105

**Published:** 2024-12-14

**Authors:** Erik A. Turner, Samantha D. Clark, Víctor Hugo Peña-García, Rebecca C. Christofferson

**Affiliations:** 1 Department of Pathobiological Sciences, Louisiana State University, Baton Rouge, LA 70803, USA; 2 Department of Biology, Stanford University, Stanford, CA 94305, USA; vhpena@stanford.edu; 3 School of Medicine, Stanford University, Stanford, CA 94305, USA

**Keywords:** microclimate, temperature, extrinsic incubation temperature, vector competence, Zika, chikungunya, *Aedes aegypti*

## Abstract

*Aedes aegypti* are indoor-dwelling vectors of many arboviruses, including Zika (ZIKV) and chikungunya (CHIKV). The dynamics of these viruses within the mosquito are known to be temperature-dependent, and models that address risk and predictions of the transmission efficiency and patterns typically use meteorological temperature data. These data do not differentiate the temperatures experienced by mosquitoes in different microclimates, such as indoor vs. outdoor. Using temperature data collected from Neiva Colombia, we investigated the impact of two microclimate temperature profiles on ZIKV and CHIKV infection dynamics in *Ae. aegypti*. We found that the vector mortality was not significantly impacted by the difference in temperature profiles. Further, we found that the infection and dissemination rates were largely unaffected, with only ZIKV experiencing a significant increase in infection at outdoor temperatures at 21 days post-infection (dpi). Further, there was a significant increase in viral titers in the abdomens of ZIKV-infected mosquitoes at 21 dpi. With CHIKV, there was a significant titer difference in the abdomens of mosquitoes at both 7 and 14 dpi. While there were differences in vector infection kinetics that were not statistically significant, we developed a simple stochastic SEIR-SEI model to determine if the observed differences might translate to notable differences in simulated outbreaks. With ZIKV, while the probability of secondary transmission was high (>90%) under both microenvironmental scenarios, there was often only one secondary case. However, CHIKV differences between microenvironments were more prominent. With over 90% probability of secondary transmission, at indoor conditions, the peak of transmission was higher (over 850 cases) compared to the outdoor conditions (<350 cases). Further, the time-to-peak for indoor was 130 days compared to 217 days for outdoor scenarios. Further investigations into microenvironmental conditions, including temperature, may be key to increasing our understanding of the nuances of CHIKV and ZIKV vectorial capacity, epidemiology, and risk assessment, especially as it affects other aspects of transmission, such as biting rate. Overall, it is critical to understand the variability of how extrinsic factors affect transmission systems, and these data add to the growing catalog of knowledge of how temperature affects arboviral systems.

## 1. Introduction

Chikungunya (CHIKV) and Zika (ZIKV) viruses are global public health threats. Both were originally isolated in Africa, but since then, they have spread across the globe [1,2,3,4,5,6,7]. In 2014, CHIKV emerged in the Americas on the Island of Saint Martin, and the subsequent epidemic included over 50 territories and over 1 million cases [8,9]. Symptomology includes high fever, maculopapular rash, arthralgia, and myalgia [10], with severe cases resulting in long-term arthralgia lasting up to 36 months [11,12]. CHIKV has since become endemic to the South American and Caribbean regions, with over 200,000 cases and 87 deaths occurring in 2022 [13,14]. Soon after, ZIKV was first identified in the Western Hemisphere in Brazil in 2015 [15]. Over the next year, more than 1.5 million cases of ZIKV occurred in South America and the Caribbean [16,17,18], and it was during this epidemic that a correlation between ZIKV infection and two sequelae, Guillain–Barré syndrome and Congenital Zika Syndrome (CZS), was first associated with infection [7,19]. Case numbers have decreased since 2016, but low levels of transmission in South America continue through 2023 [13,20].

In the tropical regions of Africa and Asia, CHIKV re-emerged between 2005 and 2006, respectively (reviewed in [9]). In particular, in 2007, a large outbreak in Sri Lanka resulted in 37,000 suspected cases [21]. The most recent reported outbreak of CHIKV in Africa was in the Republic of Congo in 2019 [22]. There are less data regarding ZIKV in these areas [23], though circulation was detected via serology in West Africa [24]. In Oceania, the first major outbreak was noted on the Yap Islands in 2007, with over 5000 cases. More recent circulation of the virus has been observed in Micronesia and the Philippines [25,26].

In the last few years, autochthonous transmission of these viruses has been observed in less tropical areas. In 2007, CHIKV emerged in Italy, where over 220 cases were confirmed, and 25 individuals were hospitalized [27]. A decade later, another outbreak in Central Italy resulted in 270 confirmed cases [28]. In 2010, two local transmission cases of CHIKV were noted in France, another 12 in 2014, and 15 confirmed cases in 2017 [27,28]. Similarly, the first local transmission of ZIKV in Europe occurred in 2017 in France [29]. During 2016–2018, local ZIKV transmission was observed in Northern Argentina [30]. This indicates that ZIKV and CHIKV are very relevant arboviruses with expanding distributions and considerable potential for detrimental effects on public health and the economy of affected regions [23].

Both viruses are primarily transmitted within urban cycles via *Aedes aegypti*, though *Aedes albopictus* has been identified as a competent vector for both [31,32]. Temperature is a dynamic environmental property, varying in range depending on climate zone, shade cover, types of ecologies, urban sprawl, and many other factors. Mosquitoes, as poikilotherms, are heavily influenced by ambient temperature, which, in turn, can impact arbovirus transmission. Mosquito lifespan, biting patterns, vector competence, and the extrinsic incubation period (EIP) are all altered by temperature [33,34,35,36,37,38,39]. Vector competence, for instance, generally increases with temperature (reviewed in [40,41]), specifically with ZIKV and CHIKV [35,42]. The extrinsic incubation period (EIP)—the time it takes for a mosquito to be able to transmit given exposure to a virus—is also known to be temperature-dependent, generally decreasing as temperature increases [35,36]. Additionally, mosquito mortality is also temperature-dependent in a non-monotonic manner [43,44,45].

While thermal curves from average temperature experiments are invaluable, a more nuanced view of temperature as a factor for arbovirus transmission is to include diurnal temperature ranges (DTR). Previous works have found that DTRs also acted on arboviral transmission systems, even when daily averages were not very different. For example, when comparing different DTRs around a common mean temperature, research found that vector competence was affected in proportion to the scale of the DTR, with larger DTRs about the mean resulting in lower vector competence rates [46,47].

Temperatures and/or DTRs derived from local weather stations are often used to inform studies investigating the impact of temperature on arbovirus transmission. However, an important consideration for the impacts of temperature on arbovirus transmission is microclimate (or microenvironment). This is especially true of ZIKV and CHIKV transmission as *Ae. aegypti* is the primary vector, and these mosquitoes tend to live within or right outside of dwellings [48]. Furthermore, *Ae. aegypti* collected inside households were found to be more likely to be involved in arbovirus transmission than mosquitoes collected from outside the domiciles [49,50]. Thus, while outside-derived temperatures have been successfully used to demonstrate the temperature-dependent phenomena, indoor microclimate is potentially a more accurate representation of what an *Ae. aegypti* mosquito experiences.

Microclimate as a factor in disease transmission has been explored experimentally in a few transmission systems. Perhaps the most explored systems fall in the agricultural realm, as greenhouses provide controllable climates to study various fungal and bacterial diseases, as well as pest arthropods that damage crops [51]. In the sphere of arthropod-borne diseases, the impact of microclimates has increased the transmission potential of malaria via increased survival rates and shortened gonotrophic cycles [52,53,54]. For arboviruses, modeling has been utilized as a tool for estimating the impacts of microclimate on transmission potential. Boser et al. used remote sensing data to model favorable temperatures for *Cx. tarsalis* in California to infer that West Nile Virus risk might change from agricultural zones to urban zones at different periods of the day due to microclimates [55]. Field-collected temperature data have been used to further divest microclimates from meteorological data to generate models for the extrinsic incubation period (EID) that match disease outbreaks of arthropod-borne viruses in Denmark outside of typical seasonality [56]. However, experimental investigations into the effects of microclimate on mosquito-borne viruses, particularly within vector kinetics, remain underexplored.

Data collected from Colombia by the Christofferson Lab demonstrated that the temperature in household microenvironments differed from those directly outside the same domiciles [57]. Given that CHIKV and ZIKV are now endemic in Colombia [58,59], understanding the impact of microclimate on within-mosquito viral dynamics is important for understanding the role of microenvironmental temperature on *Ae. aegypti*-borne virus transmission. Herein, we explore the hypothesis that Colombian microclimates affect the within-host dynamics of ZIKV and/or CHIKV.

## 2. Materials and Methods

*Derivation of Colombian temperature profiles:* Temperatures from five houses in Neiva, Colombia, were collected every four hours each day, from both inside and outside of the houses from 29 July 2019 to 21 April 2020 [57]. The placement of HOBO digital data loggers in the house was based on personal communication of inhabitants on where they encountered the most mosquitoes; outside loggers were placed either on the veranda, directly outside the door, or on the nearest outdoor structure [57].

**Figure 1:**
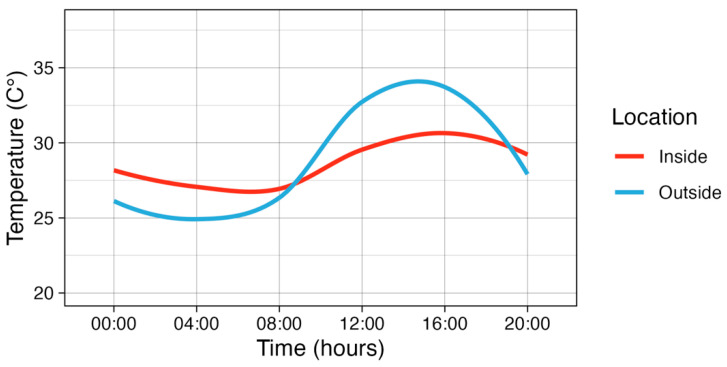
Temperature profile generated for Neiva, Colombia, based on inside or outside microclimate sampling from [52].

The months of January and December were determined to have the most arbovirus cases in Neiva according to the Colombian national public health surveillance system, Sistema Nacional de Vigilancia en Salud Pública (data from years 2011–2020). Two profiles were generated based on January–December temperature loggers [57] for inside and outside microclimates (Figure 1), and environmental chambers were programmed to each microclimate. The DTRs for the inside profile was 3.5 °C (average: min = 26.8 °C; max = 30.3 °C; mean = 28.4 °C) and 8.2 °C (min = 24.7 °C; max = 32.9 °C; mean = 28.0 °C) (Figure 1) for outside. Humidity was consistently maintained at approximately 65% per average historical humidity in the area, as determined by an average humidity from 2017 to 2022 for December per Weather Underground [60]. Temperature and humidity within the environmental chambers were monitored via Kestral Drop 2 temperature loggers, recording data every ten minutes. These temperature profiles defined the extrinsic incubation temperature (EIT) for inside vs. outside treatments.

*Viruses and mosquitoes:* CHIKV was obtained from the World Arbovirus Reference Center at the University of Texas Medical Branch. The CHIKV strain SM2013 was originally isolated from a human patient in St. Martin in 2013 and had a passage history of P2J3V2 at receipt. Subsequently, the stock was passed twice more through Vero E6 cells. The ZIKV strain, PRVABC59 (Asian lineage), was isolated from serum in Puerto Rico in 2015 from a human patient and was provided by Dr. Barbara Johnson at the US Centers for Disease Control and Prevention. *Ae. aegypti* (Rockefeller colony) were vacuum-hatched for 45 min in ddH_2_O and reared until pupation at 28 °C. Larva were given ground fish food ad libitum. Pupae were removed, counted, and relocated to environmental chambers programmed for one of the two temperature profiles (inside vs. outside) within the BSL-3 laboratory at the LSU School of Veterinary Medicine.

*Viral RNA detection and quantification*: Quantification of viral RNA was performed via qRT-PCR post-viral RNA extraction via MagMax 96 viral isolation, as previously described in [61]. qRT-PCR was performed on a Roche Lightcycler96 using Quantabio Ultratough Mastermix. For ZIKV, the primers targeted the NS5 region [62,63,64], and for CHIKV, the E3 region was targeted [65]. For each extraction and subsequent assay, a standard curve was performed and tied to a crystal violet plaque assay in order to quantify viral RNA [66].

*Oral exposure of Ae. aegypti to CHIKV or ZIKV*: Adult female mosquitoes 3–5 days post-emergence were offered an infectious bloodmeal composed of 2:1 bovine blood in Alsevers (Hemostat Labs, Dixon, CA, USA) to infectious supernatant containing either CHIKV or ZIKV via a Hemotek feeding apparatus (Discovery Labs, Blackburn, UK). Mosquitoes were sugar-starved for 24 h prior to exposure. For ZIKV, the infectious titer of the proffered bloodmeal ranged from 1.12 to 5.73 × 10^5^ pfu/bloodmeal. For CHIKV, the infectious bloodmeals ranged from 3.67 to 5.17 × 10^5^ pfu/bloodmeal. Following feeding, engorged females were cold-anesthetized, counted, and placed into clean cartons. Two biological replicates were performed for each virus/temperature combination. Each container of females was offered 10% sucrose ad libitum and a wetted oviposition paper, which was checked daily and re-wetted if necessary.

*Vector competence of Ae. aegypti for CHIKV or ZIKV*: At 7, 14, and 21 days post-infectious bloodmeal, females were sampled and assessed for midgut infection, disseminated infection, and transmission [67]. Briefly, mosquitoes were cold-anesthetized, and the legs and wings were removed. Legs and wings were collected in locking tubes filled with BA-1 media and two stainless steel BBs. Mosquitoes were then immobilized on double-sided tape, and the proboscis was inserted into a 20 µL pipette tip containing 35 nM ATP in 35 µL of fetal bovine serum to stimulate salivation. After 30 min, the mosquito’s body was placed into a tube containing BA-1 and two stainless steel BBs. Mosquito tissue samples were then homogenized (Qiagen Tissuelyzer) and stored at −80 °C until processing. The salivation solution was decanted into a tube and likewise stored at −80 °C until processing.

*Ae. aegypti mortality in response to different temperature profiles*: An additional cohort of *Ae. aegypti* were hatched and reared as larvae at 28 °C before being transferred to environmental chambers with the Colombian inside and outside temperature profiles as pupae. At 3–5 days post-emergence, a non-infectious bloodmeal was offered for 45 min. Then, mosquitoes were sorted as described above and placed back in environmental chambers at their respective temperature profiles. Two biological replicates (cartons) were performed at each temperature profile (inside vs. outside). As mentioned above, each carton was offered 10% sucrose ad libitum and wetted oviposition paper. Mosquitos that died prior to 2 days post-bloodmeal were censored from the study (*n* = 2). Mosquito mortality was recorded daily for 21 days post-bloodmeal.

*Statistical analysis*: The probability of mosquito survival at each timepoint was determined via a Kaplan–Meier survival curve using the ggsurvfit and survival libraries in R studio [68]. To test for differences in mortality rates among treatments, Kaplan–Meier survival analyses were conducted, and the average time to death (TTD) was estimated for each temperature profile. To test for a difference in the proportion of mosquitoes that tested positive for infection, dissemination, or transmission between the inside and outside mosquitoes, a Chi-squared test of homogeneity was utilized using the prop.test function. A Kruskal–Wallace non-parametric analysis of variance was applied to examine differences in titers of Viral RNA recovered from mosquito abdomens, legs/wings, or saliva. Differences were assessed across the three-sampled days post-exposure between the inside and outside temperature profiles (post-hoc Dunn’s test, Bonferroni). On days where all data were 0 (i.e., no variability) for one group but not another, a one-sample Mann–Whitney test was used to determine whether the group with data was significantly different from the null value of zero, thus acting as a proxy comparison for the group with zero-values. Significance was assessed at α= 0.05.

*Modeling transmission potential of CHIKV and ZIKV* in *Ae. aegypti with observed differences:* Recently, it was demonstrated that disseminated infection may be a better metric for determining the transmission potential of arboviruses in *Ae. aegypti* [69]. Thus, we used dissemination as a proxy to parameterize vector competence in a stochastic SEI-SEIR model (see Supplemental Information for model equations and transition rates). This exercise is conducted to show that while results might not be statistically significant, there is the potential for some observable biological effect. Data from the experimental investigations were used to parameterize the probability of transmission from human to mosquito, the probability of transmission from mosquito to human, and the probability of daily survival. The probability of transmission from human to mosquito was derived from the maximum proportion of successful infections in the midgut following exposure [33,57,67]. The probability of transmission from mosquito to human was parameterized using the values of vector competence at 14 days post-exposure. Other parameter values were mined from the literature, such as the average intrinsic incubation period of ZIKV (6.2 days) and CHIKV (3 days) [70,71], as well as the infectious period of humans for ZIKV (r^−1^ = 5 days) and CHIKV (r^−1^ = 6) [72,73]. To isolate the effects and compare the proportionality of temperature-dependent parameters measured in this study, the biting rate was kept at once daily; the mosquito density was kept constant with an emergence rate of 5000 female adult mosquitoes every 7 days, and the human population was kept stable at 38,000 individuals, with no mortality or birth into the population [74]. The model was run for 500 realizations over the course of 365 days with the following assumptions, using the tau-leap approximation to Gillespie’s algorithm [30,74]. There was no explicit spatial or temporal definition in the model parameters, and there was an assumption of homogenous mixing. A time-step of 0.125 days was used with model output occurring 1/day.

## 3. Results

**Figure 2:**
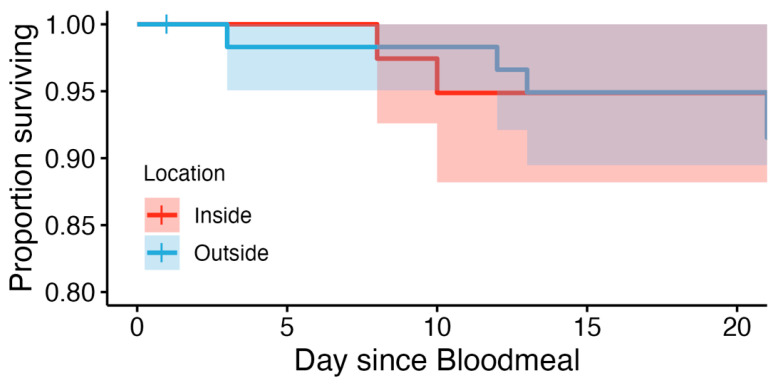
Kaplan–Meier survival curve of *Ae. aegypti* mosquitoes held at temperature profiles associated with inside (inside: red) or outside (outside: blue) microenvironments.

*Ae. aegypti mortality in response to different temperature profiles:* Replicates were combined after determining no statistical difference between them. Thus, the total sample sizes were *n* = 40 for inside and *n* = 60 for outside treatments. For both the inside and outside groups, the probability of *Ae. aegypti* survival at each timepoint in the mortality study was high (Figure 2). At the inside microclimate, the probability of survival was 100% at 7 days post-bloodmeal, and 94.9% (95% confidence interval 88.2–100%) at both 14 and 21 days post-bloodmeal. The mosquitoes in the outside microclimate had survival probabilities of 98.3% (CI: 95.1–100%), 94.9% (CI: 89.5–100%), and 91.5% (CI: 84.7–98.9%) to survive at 7, 14, and 21 days post-bloodmeal, respectively. However, differences were not statistically significant (*p* = 0.5). The probability of daily survival was estimated to be 94.9% for both temperature profiles.

**Figure 3:**
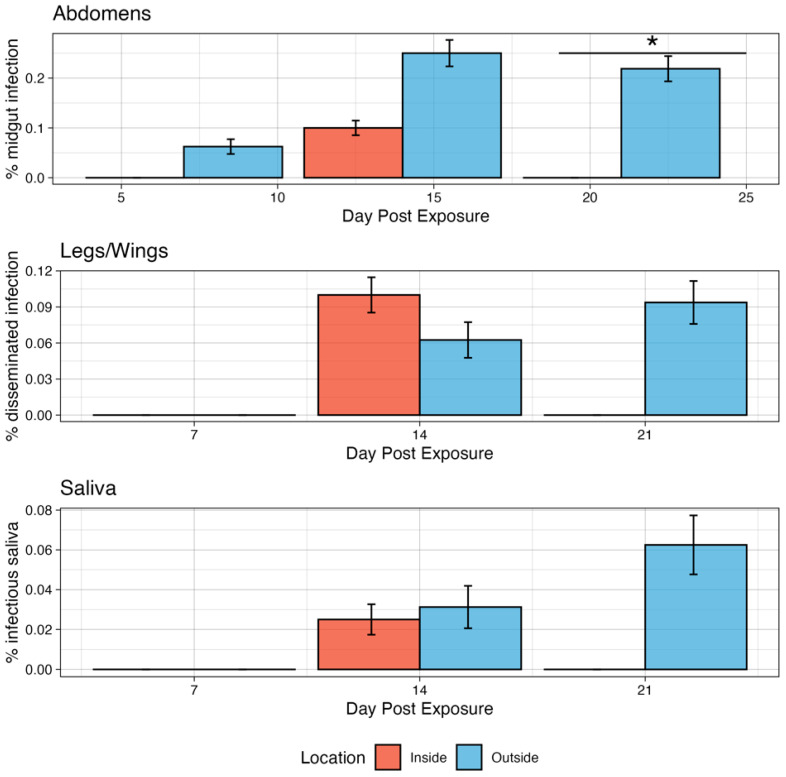
Infection kinetics of ZIKV in *Ae. aegypti* mosquitoes demonstrating the % that developed a midgut infection in the abdomen, disseminated infection in the legs/wings, and had virus in the saliva. Error bars are 95% binomial confidence intervals. * indicates *p* < 0.05.

*Infection kinetics of Ae. aeygpti for ZIKV under two microclimate conditions:* At both microclimates, *Ae. aegypti* developed midgut infections for ZIKV (Figure 3), though to modest levels. At 7 dpi, two (6.25%) mosquitoes in the outside scenario were infected, while none in the inside conditions were infected, though this was not significant (*p* = 0.3989). On day 14, 10% of mosquitoes had developed a midgut infection for the inside condition compared to 25% in the outside condition, though, again, this was not significant (*p* = 0.1679). On day 21, none of the mosquitoes tested for the inside condition were positive, while 21.9% on the outside were positive, and this was significant (*p* = 0.02043). There was no significant difference in the inside temperature profile among timepoints.

Next, the dissemination of infection to the legs and wings was examined. Neither the inside nor the outside conditions had disseminated infections on day 7 dpi. On day 14, 10% of inside and 6.25% of outside mosquitoes were positive for disseminated infection, though this was not significant (*p* = 0.8863). On day 21, no mosquitoes tested for inside had disseminated infections, while 9.38% had disseminated infection in the outside group, though, again, this was not significant (*p* = 0.2064). Only one mosquito in the inside group had detectable virus in the saliva at 14 dpi, and the overall transmission efficiency was 25% (1/4). In the outside group, there was detectable virus in the saliva of one and two mosquitoes on days 14 and 21, respectively. The median abdomen titer of ZIKV-infected mosquitoes was 0 on days 7 and 21 for the inside group and 4.93 Log10 (PFU/mL) on day 14 post-exposure.

**Figure 4:**
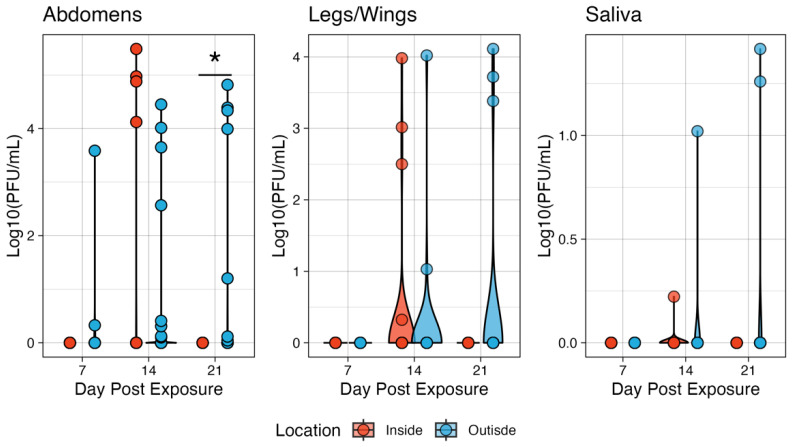
The ZIKV viral titers in the tissues of *Ae. aegypti* mosquitoes at two microenvironmental conditions. The body titers of the inside group on day 14 were significantly different from those of the outside group. Titers were analyzed on a non-log scale but are presented on a log scale for visualization. * indicates *p* < 0.05.

The outside group had higher titers of median 1.96, 1.49, and 3.99 Log10 (PFU/mL) at 7, 14, and 21 days post-exposure, respectively (Figure 4). The only significant difference in titer between microclimates was on day 14, when the inside group had a higher median titer compared to the outside group. The outside group abdomen titers on days 7 and 21 were not statistically significantly different from a null value of 0. Similarly, there was little effect on dissemination rates. On day 7 post-exposure, neither condition had mosquitoes with a disseminated infection. On day 14, the median titer of legs and wings was 2.76 and 2.52 Log10 (PFU/mL) for inside and outside conditions, respectively. Given the low number of mosquitoes with virus detectable in the saliva, statistical testing was not appropriate, though in general, the outside group had higher titers on day 14 post-exposure with a median saliva titer of 1.02 Log10 (PFU/mL) compared to 0.222 Log10 (PFU/mL), though this represents an *n* = 1 for each group. On day 21, two mosquitoes in the outside group had viruses in the saliva with titers of 3.72 and 4.11 Log10 (PFU/mL) (Figure 4).

**Figure 5:**
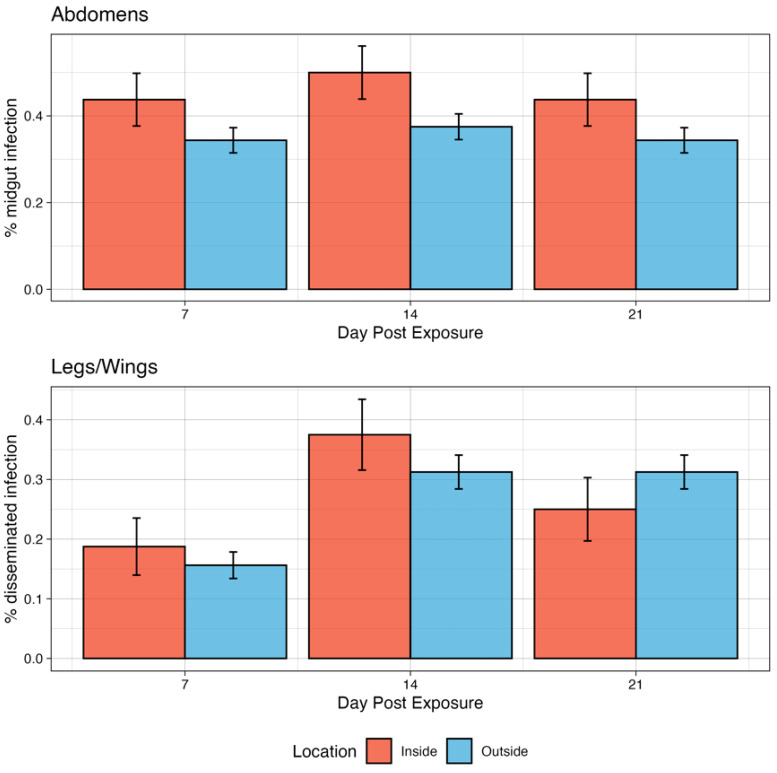
Infection kinetics of CHIKV in *Ae. aegypti* mosquitoes demonstrating the % that developed a midgut infection in the abdomen and disseminated infection in the legs/wings. Error bars are 95% binomial confidence intervals.

*Infection kinetics of Ae. aeygpti for CHIKV under two microclimate conditions:* By day 7 dpi, 43.8% of exposed mosquitoes in the inside group had developed a midgut infection (Figure 5). This increased modestly to 50% on day 14 dpi and then decreased again to 43.8% on day 21 dpi. There were no significant differences in infection rates among timepoints. Similarly, for the outside group, 34.4% of exposed mosquitoes were infected by 7 dpi, rising modestly to 37.5 at 14 dpi and then to 34.4% at 21 dpi. There were no significant differences in infection rates among timepoints. Though the inside group was modestly higher in infection rates compared to the outside group at all timepoints, this was not statistically significant (*p* > 0.05).

Disseminated infections in the legs and wings were detected at all timepoints for CHIKV at both inside and outside profiles (Figure 5). At the inside profile, the dissemination rate increased from 7 dpi (18.8%) to 14 dpi (37.5%) but decreased slightly from 21 dpi to 25% (*p* = 0.9106). However, these differences were not significant. At 7 dpi, 15.6% of exposed mosquitoes had developed a disseminated infection in the outside group, which rose to 31.2% on day 14 and remained unchanged on day 21. These differences were also not significant. When comparing the two conditions on each day post-infection, there were no significant differences between the inside vs. outside groups. There were no mosquitoes that had detectable virus in the saliva for either microenvironmental group exposed to CHIKV.

**Figure 6:**
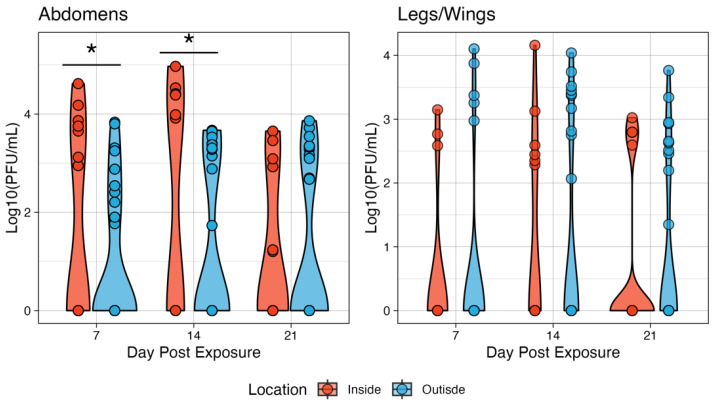
The CHIKV viral titers in the tissues of *Ae. aegypti* mosquitoes at two microenvironmental conditions. The body titers of the inside group on days 7 and 14 were significantly different from those of the outside group. Titers were analyzed on a non-log scale but are presented on a log scale for visualization. Simulating the effects of Differential Within-Vector Factors on population-level transmission. A transmission model was parameterized using our experimental data for each of the viruses and locations (Table 1). * indicates *p* < 0.05.

There was a significant effect of microclimate on the infection titers of CHIKV-exposed *Ae. aegypti*. On Day 7, the inside group had overall higher titers compared to the outside group (Figure 6), with medians of 3.75 and 2.54 Log10 (PFU/mL), respectively (*p* = 0.0164). Similarly, the inside conditions produced higher titers on day 14 post-exposure, with median titers of 4.40 and 3.39 Log10 (PFU/mL), respectively (*p* = 0.0002). However, on day 21, there were no significant differences observed in titer (medians of 3.09 and 3.33 for inside vs. outside, respectively). With respect to dissemination, there were no significant differences in titers when compared between the two microclimates. The median titer on day 7 was 2.77 Log10 (PFU/mL) inside and 3.37 Log10 (PFU/mL) outside. On day 14, the inside group reached a median titer of 2.52 Log10 (PFU/mL) and 3.38 outside. Finally, on day 21, there was a median titer of 2.80 inside and 2.64 outside (Figure 6).

**Figure 7:**
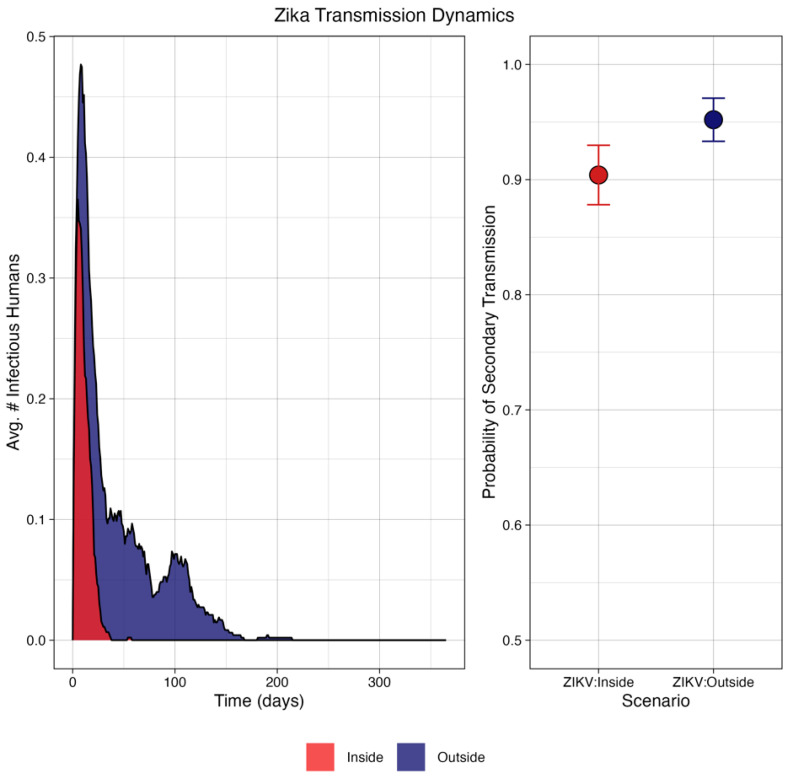
Simulation of transmission dynamics of ZIKV under conditions defined by microenvironment showing (**left**) the average epidemic curve, which failed to produce fulminant outbreaks; and (**right**) the probability of at least one secondary human infection was approximately the same under both conditions: 90.4% inside, and 95.2% outside. Error bars represent binomial 95% confidence intervals.

It was found that, in general, the ZIKV virus was not likely to propagate under either scenario, reaching a maximum of one secondary case in any simulation. However, there was a high probability of that secondary case (Figure 7) at 90.4% for the inside condition and 95.2% for the outside condition.

**Figure 8:**
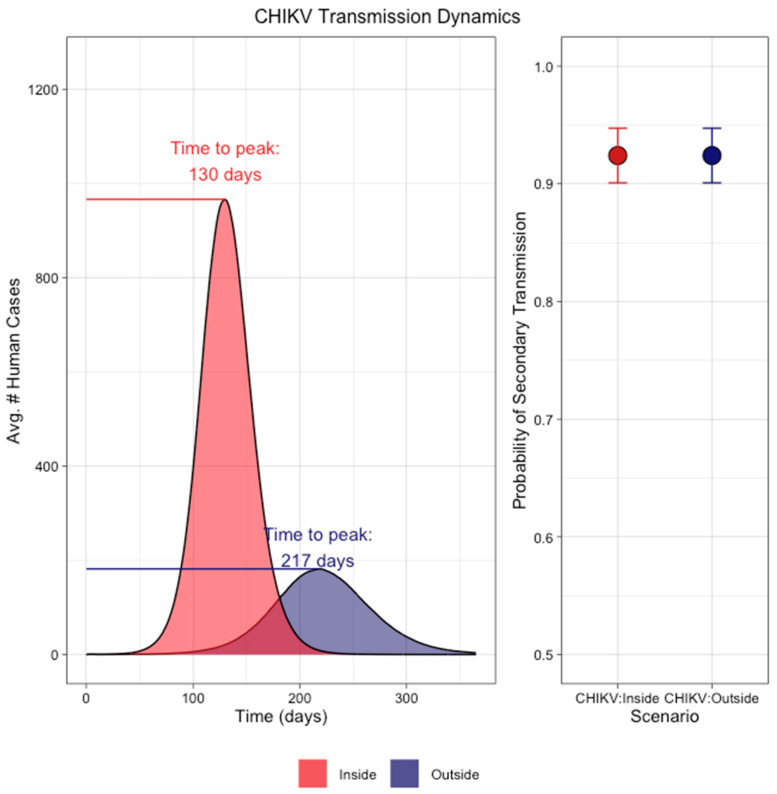
Simulation of transmission dynamics of CHIKV under conditions defined by microenvironment showing (**left**) the average epidemic curve under inside (red) and outside (blue) conditions. The dissemination patterns under indoor temperature conditions produced on average over 57,000 total cases while the outside temperature conditions resulted in approximately 19,000 human cases. The difference in time-to-peak was 87 days between the two conditions. (**Right**) The probability of at least one secondary human infection was approximately equal under the two conditions (92.4%). Error bars represent binomial 95% confidence intervals.

CHIKV transmission using dissemination as a proxy was more established under both microenvironments (Figure 8). There was a noticeable difference in the magnitude of cases resulting from the two different microenvironments. The CHIKV dynamics parameterized from the outside conditions had an average total cumulative case count of 19,873 cases, while the inside conditions reached 57,148 total cases. In addition, the average time to peak was less in the inside scenario at 130 days, while the time to peak was 217 days on average for the outside scenario. In both cases, there was a high probability (92.4%) of at least one secondary case arising.

## 4. Discussion

Following the explosive nature of their emergences in South America, CHIKV and ZIKV are now endemic in Colombia, as low levels of transmission have continued to occur annually [8,9,16,17,18,58,59]. The predicted epidemiology of these viruses informs public health preparedness and responses before and during outbreaks to best serve the public and reduce disease burden. Many processes that drive transmission, especially within the mosquito vector, are affected by temperature [33,34,35,36,37,38,39]. Many predictive models use outdoor temperature readings to inform computational models, as well as experimental conditions that feed those models. The primary vector of CHIKV and ZIKV in South America is *Ae. aegypti*, [31,32], and this species lives and, therefore, transmits virus within domiciles [49,50]. Thus, the impact of microclimates, specifically the differences between indoor and outdoor temperature profiles, is important for understanding the whole transmission puzzle. Indeed, others have demonstrated that microclimate is an important factor in arbovirus transmission [75,76], and microclimate has been indicated in differences in malaria transmission [77].

These data demonstrate that accounting for microclimate can demonstrate observable differences in the mosquito viral kinetics of both viruses, though this was not statistically significant in most cases. However, on day 21 dpi, there was a significant difference in the infection rates of ZIKV-exposed mosquitoes. A single day’s significant difference in viral kinetics was determined to be the driver of CHIKV adaptation to *Ae. albopictus* [78]. Thus, in Neiva, there is the potential that this small difference in the infection rate of ZIKV (where outdoor conditions are higher than indoor) may significantly affect estimates of transmission risk. Interestingly, no effect was observed for CHIKV, though competence for the virus remained moderately high in both microclimates, indicating an overall fitness in *Ae. aegypti*. However, it is important to note that these colony mosquitoes may not represent the population dynamics of Colombian mosquitoes, as population differences in vector competence are known to exist [79].

In addition to infection and dissemination rates, differences in viral titer in infected mosquito midguts and peripheral tissues were observed. There are mixed data on the importance of viral titer in the midgut in regards to midgut barrier escape, as reviewed in [80]. The only significant difference in titer observed in this study was between CHIKV-exposed mosquitoes at 14 dpi, with a higher titer observed in inside than outside mosquitoes. This did not correspond to a higher proportion of disseminated infections at the same timepoint or at the 21 dpi time point.

It is interesting that this effect was observed for ZIKV and not CHIKV in terms of infection and dissemination rates. This could be related to how temperature acts on viral particles themselves. Additionally, temperature can influence the binding affinity of the viral glycoproteins and cell receptors for CHIKV, DENV, and other arboviruses [81]. Both interactions are sensitive, indicating that the smaller DTR changes between the microclimates may affect mosquito infection via these and similarly sensitive factors.

In the simulation study, the trends in successful secondary transmissions were less for ZIKV under indoor conditions, while no difference was noted between the two locations for CHIKV. Further, CHIKV under indoor conditions resulted in a higher magnitude of cases compared to outdoor conditions. This is particularly interesting, as it points to a virus–vector-specific interaction and argues against the generalization of temperature trends across systems.

Further investigations into microenvironmental conditions, including temperature, may be key to increasing our understanding of the nuances of CHIKV and ZIKV vectorial capacity, epidemiology, and risk assessment, especially as it affects other aspects of transmission, such as biting rate [33]. Understanding the role of microclimate across arbovirus systems would improve model inferences and inform predictions, especially in the context of climate change and the role of climate infrastructure (e.g., air conditioning) as a means to combat transmission. Overall, it is critical to understand the variability of how extrinsic factors affect transmission systems, and these data add to the growing catalog of knowledge of how temperature affects arboviral systems.

## Figures and Tables

**Table 1:** Parameter values derived from experimental data used to model transmission of CHIKV or ZIKV at two microclimate conditions, temperature profiles of which are given in Figure 1.

Parameter	Virus	Location	Value
Successful transmission from human to mosquito (beta)	ZIKV	Inside	3.3%
		Outside	18.0%
	CHIKV	Inside	46.0%
		Outside	35%
Vector Competence/EIP	ZIKV	Inside	10% at 14 dpi
		Outside	6.25% at 14 dpi
	CHIKV	Inside	75% at 14 dpi
		Outside	31.2% at 14 dpi
Probability of daily survival	Both	Inside	0.949
		Outside	0.949

## Data Availability

All data are contained in the manuscript or supplemental information.

## Supplementary Materials

Supplemental Information can be downloaded at: https://www.mdpi.com/article/10.3390/pathogens13121105/s1, Table S1: Transition rates between compartments of the SEI-SEIR model.
