# Supplementary material for: Investigating the Effects of Microclimate on Arboviral Kinetics in Aedes aegypti
Source: Pathogens. 2024 Dec 14;13(12):1105. doi: 10.3390/pathogens13121105 (PMC11728849; doi:10.3390/pathogens13121105)
Supplement: Supplementary file 1 [file pathogens-13-01105-s001.zip › pathogens-3337794-supplementary.pdf]

Supplemental Information:
Turner et al.
Exploring the role of microclimate for arbovirus infection dynamics

Human Side Equations:

$$\frac{dS_h}{dt} = S_h - aS_h\frac{I_m}{N_h}$$

$$\frac{dE_h}{dt} = a * S_h * \frac{I_m}{N_h} - \varpi E_h$$

$$\frac{dI_h}{dt} = \varpi E_h - \ vI_h$$

$$\frac{dR_h}{dt} = R_h + \ vI_h$$

Mosquito Side Equations

$$\frac{dS_m}{dt} = \varepsilon - aS_m\frac{\vartheta I_h}{N_h} - \mu S_m$$

$$\frac{dE_m}{dt} = aS_m * \frac{\vartheta I_h}{N_h} - \chi\beta E_m - \mu E_m$$

$$\frac{dI_m}{dt} = \chi\beta E_m - \mu I_m$$

Table S1: Transition rates between compartments of the SEI-SEIR model

Event	Change in state	Transition rate
Transmission from mosquito to human	$(S_h,E_h) \rightarrow (S_h-1, E_h+1)$	$aS_h\frac{I_m}{N_h}$
Onset of infectiousness in human	$(E_h,I_h) \rightarrow (E_h-1, I_h+1)$	ϖE_h
Recovery in human	$(I_h,R_h) \rightarrow (I_h-1, R_h+1)$	vI_h
Adult (female) mosquito recruitment	$(S_m) \rightarrow (S_m+1)$	ε
Susceptible mosquito death	$(S_m) \rightarrow (S_m-1)$	μS_m

Event	Change in state	Transition rate
Transmission from human to mosquito	$(S_m, E_m) \rightarrow (S_m - 1, E_m + 1)$	$aS_m \frac{\vartheta I_h}{N_h}$
Exposed mosquito death	$(E_m) \rightarrow (E_m - 1)$	μE_m
Onset of infectiousness in mosquito	$(E_m, I_m) \rightarrow (E_m - 1, I_m + 1)$	$\chi \beta E_m$
Infectious mosquito death	$(I_m) \rightarrow (I_m - 1)$	μI_m

Parameter definitions:

a – biting rate (held constant at 1)
ϖ^{-1} - the intrinsic incubation period of each virus in the human
ν^{-1} - the recovery rate of humans for each virus
ε - the emergence rate of female mosquitoes
μ - the probability of daily survival for adult female mosquitoes
ϑ – the probability of successful transmission from human to mosquito given a bite
β^{-1} - the extrinsic incubation period of the virus in the mosquito
χ - the probability of successful transmission from the mosquito to the human (vector competence)
